# Supplementary material for: Population structure and phylogenetic analysis of Vibrio cholerae non-O1/O139 by whole genome sequencing
Source: PLoS One. 2026 Mar 5;21(3):e0343306. doi: 10.1371/journal.pone.0343306 (PMC12962490; doi:10.1371/journal.pone.0343306)
Supplement: Attachment — Submitted filename: Response to Reviewers.docx [file pone.0343306.s004.docx]

**Response to Reviewers**

Dear Editor and Reviewers,

Thank you for the thorough review and constructive feedback you provided on my manuscript submitted to PLOS ONE. Your insightful comments have been invaluable in improving the quality and clarity of the manuscript. I have carefully considered each of your suggestions. Below are the responses point-by-point to journal requirements and reviewer’s feedback. We hope our revised version meets your expectations.

Thank you once again for your time, effort, and valuable contributions to the advancement of this work.

Best regards,

Taylor Wells

Corresponding Author

[taylor.m.wells@phac-aspc.gc.ca](mailto:taylor.m.wells@phac-aspc.gc.ca)

PONE-D-25-23191

| **Journal requirements** | **Author corrections** |
| --- | --- |
| 1. Please ensure that your manuscript meets PLOS ONE's style requirements, including those for file naming. The PLOS ONE style templates can be found at  <https://journals.plos.org/plosone/s/file?id=wjVg/PLOSOne_formatting_sample_main_body.pdf> and  <https://journals.plos.org/plosone/s/file?id=ba62/PLOSOne_formatting_sample_title_authors_affiliations.pdf> | Thank you. The instructions of the manuscript body formatting guidelines and title, author,  affiliations formatting guidelines have been thoroughly reviewed and followed, achieving compliance with the style requirements of PLOS ONE, including those of file naming. |
| 2. Thank you for uploading your study's underlying data set. Unfortunately, the repository you have noted in your Data Availability statement does not qualify as an acceptable data repository according to PLOS's standards.    At this time, please upload the minimal data set necessary to replicate your study's findings to a stable, public repository (such as figshare or Dryad) and provide us with the relevant URLs, DOIs, or accession numbers that may be used to access these data. For a list of recommended repositories and additional information on PLOS standards for data deposition, please see<https://journals.plos.org/plosone/s/recommended-repositories>. | We have listed our GenBank repository which is a valid data repository according to PLOS’s standards. Page 7 of the pdf submission states: “All sequence assemblies are available from the NCBI GenBank database and BioSample accession numbers are listed in S1 Table. Newly sequenced genomes are available under NCBI’s BioProject PRJNA1230206. All other relevant data are within the manuscript and its Supporting Information files.” |
| 3. We note that Figure 1 in your submission contain map images which may be copyrighted. All PLOS content is published under the Creative Commons Attribution License (CC BY 4.0), which means that the manuscript, images, and Supporting Information files will be freely available online, and any third party is permitted to access, download, copy, distribute, and use these materials in any way, even commercially, with proper attribution. For these reasons, we cannot publish previously copyrighted maps or satellite images created using proprietary data, such as Google software (Google Maps, Street View, and Earth). For more information, see our copyright guidelines:<http://journals.plos.org/plosone/s/licenses-and-copyright>.    We require you to either present written permission from the copyright holder to publish these figures specifically under the CC BY 4.0 license, or remove the figures from your submission:    a. You may seek permission from the original copyright holder of Figure 1 to publish the content specifically under the CC BY 4.0 license.    We recommend that you contact the original copyright holder with the Content Permission Form (<http://journals.plos.org/plosone/s/file?id=7c09/content-permission-form.pdf>) and the following text:  “I request permission for the open-access journal PLOS ONE to publish XXX under the Creative Commons Attribution License (CCAL) CC BY 4.0 (<http://creativecommons.org/licenses/by/4.0/>). Please be aware that this license allows unrestricted use and distribution, even commercially, by third parties. Please reply and provide explicit written permission to publish XXX under a CC BY license and complete the attached form.”    Please upload the completed Content Permission Form or other proof of granted permissions as an "Other" file with your submission.    In the figure caption of the copyrighted figure, please include the following text: “Reprinted from [ref] under a CC BY license, with permission from [name of publisher], original copyright [original copyright year].”    b. If you are unable to obtain permission from the original copyright holder to publish these figures under the CC BY 4.0 license or if the copyright holder’s requirements are incompatible with the CC BY 4.0 license, please either i) remove the figure or ii) supply a replacement figure that complies with the CC BY 4.0 license. Please check copyright information on all replacement figures and update the figure caption with source information. If applicable, please specify in the figure caption text when a figure is similar but not identical to the original image and is therefore for illustrative purposes only.  The following resources for replacing copyrighted map figures may be helpful:    USGS National Map Viewer (public domain):<http://viewer.nationalmap.gov/viewer/>  The Gateway to Astronaut Photography of Earth (public domain):<http://eol.jsc.nasa.gov/sseop/clickmap/>  Maps at the CIA (public domain):<https://www.cia.gov/library/publications/the-world-factbook/index.html> and<https://www.cia.gov/library/publications/cia-maps-publications/index.html>  NASA Earth Observatory (public domain):<http://earthobservatory.nasa.gov/>  Landsat:<http://landsat.visibleearth.nasa.gov/>  USGS EROS (Earth Resources Observatory and Science (EROS) Center) (public domain): [http://eros.usgs.gov/#](http://eros.usgs.gov/)  Natural Earth (public domain):<http://www.naturalearthdata.com/> | Figure 1 was constructed using software provided by Tableau Incorporated (www.tableau.com) which uses a Canadian map provided under a CC BY-4.0 license from OpenStreetMap. The copyright from OpenStreetMap allows use with proper attribution. Further detail on the copyright from OpenStreetMap may be found at www.openstreetmap.org/copyright.  Copyright attribution was added to the bottom left corner of Figure 1. |
| 4. If the reviewer comments include a recommendation to cite specific previously published works, please review and evaluate these publications to determine whether they are relevant and should be cited. There is no requirement to cite these works unless the editor has indicated otherwise. | Noted. |
| **Reviewer’s responses to questions** |  |
| *Comments to the author* |  |
| 1. Is the manuscript technically sound, and do the data support the conclusions?    The manuscript must describe a technically sound piece of scientific research with data that supports the conclusions. Experiments must have been conducted rigorously, with appropriate controls, replication, and sample sizes. The conclusions must be drawn appropriately based on the data presented.  Reviewer #1: No  Reviewer #2: Yes | We have made several small changes to improve the clarity and soundness of the manuscript. Please see responses to specific reviewer comments below. |
| 2. Has the statistical analysis been performed appropriately and rigorously?  Reviewer #1: N/A  Reviewer #2: Yes | Statistical analysis has been re-run as per reviewer #2 comment #11 and adjustments have been made to the manuscript. |
| 3. Have the authors made all data underlying the findings in their manuscript fully available?    The [PLOS Data policy](http://track.editorialmanager.com/CL0/http:%2F%2Fwww.plosone.org%2Fstatic%2Fpolicies.action%23sharing/1/010f0197fd0b1f8d-8a25f3cb-e1ea-4491-84dd-8d3a440a4b04-000000/dLg8FzZC5hK7pLh15qhg--Ye-U9X-suR2kvFIaReMaA=219) requires authors to make all data underlying the findings described in their manuscript fully available without restriction, with rare exception (please refer to the Data Availability Statement in the manuscript PDF file). The data should be provided as part of the manuscript or its supporting information, or deposited to a public repository. For example, in addition to summary statistics, the data points behind means, medians and variance measures should be available. If there are restrictions on publicly sharing data—e.g. participant privacy or use of data from a third party—those must be specified.  Reviewer #1: No  Reviewer #2: Yes | We have carefully reviewed that all data has been made available in the following locations: NCBI, Supplementary tables, and Microreact. Specific documents and links can be found within the manuscript. |
| 4. Is the manuscript presented in an intelligible fashion and written in standard English?    PLOS ONE does not copyedit accepted manuscripts, so the language in submitted articles must be clear, correct, and unambiguous. Any typographical or grammatical errors should be corrected at revision, so please note any specific errors here.  Reviewer #1: Yes  Reviewer #2: Yes | Thank you. |
| 5. Review Comments to the Author    Please use the space provided to explain your answers to the questions above. You may also include additional comments for the author, including concerns about dual publication, research ethics, or publication ethics. (Please upload your review as an attachment if it exceeds 20,000 characters) |  |
| **Reviewer #1:** Dear authors, I went through your manuscript and found some changes that must be done. Here are my suggestions- | We are grateful for your insightful comments and constructive suggestions. Our point by point responses are as follows: |
| 1. The definition of a new CAD lineage based solely on a cluster of ≥3 isolates lacks methodological rigor. A more quantitative threshold (e.g., allelic divergence) should be applied alongside phylogenetic clustering to robustly delineate novel lineages. | We are not only using clustering to define our new lineages. The method we used was established by Liang et al., 2020 (doi: 10.1128/JB.00086-20) using their cgMLST scheme - which is publicly available on PubMLST (<https://pubmlst.org/vcholerae/>). This method uses allelic differences to create phylogenetic clusters. Clustering thresholds are defined in Table 1 of the manuscript. |
| 2. While 242 Canadian isolates were analyzed, the manuscript lacks a clear rationale for focusing on Canada in a globally relevant context. Adding a comparative or translational public health perspective (e.g., impact of NOVC on coastal communities or seafood industries) would improve relevance. | Agreed. Additional rationale has been provided in the introduction (lines 115-119 marked up copy), drawing attention to the impact of climate, *Vibrio* abundance, seafood-borne illness, and the impact of traditional harvesting practices of First Nations in coastal areas of Canada. |
| 3. The study relies heavily on cgSNP analysis but does not clarify the rationale for SNP thresholds used to define lineages, outbreaks, and sublineages. The cgMLST cutoffs (e.g., 3, 40, 133, 1000 alleles) need to be referenced and justified within the context of V. cholerae evolution. | While this study does use cgSNP analysis for creating phylogenetic trees, we defined lineages using the cgMLST schema and thresholds described by Liang et al., 2020 (doi: 10.1128/JB.00086-20) and is publicly available on PubMLST (<https://pubmlst.org/vcholerae/>). This cgMLST scheme provides standardized classification in the context of *V. cholerae*- enabling defined cutoff thresholds- important for outbreak investigations. Clustering thresholds are defined in Table 1 of the manuscript. |
| 4. Environmental data (e.g., temperature, salinity, sample site type) are not quantitatively integrated. A generalized linear model or spatiotemporal regression could better reveal correlations between environmental factors and lineage emergence. | Thank you for noting this. We did not include any environmental data such as temperature, salinity and sample site type because this information was largely not available and was not within the scope of this study. But we do agree that a spatiotemporal model would be suitable for studying within-species diversity and spatiotemporal variation in *V. cholerae* distribution.  The relationship between climate and vibrio abundance has been well established in the literature. Additionally, I’ve added a couple lines on this relationship into the introduction lines 113-119 (marked up copy). |
| 5. Given the known plasticity of V. cholerae genomes, especially NOVC strains, the potential role of HGT in virulence gene dissemination (e.g., tdh, T3SS, VSP islands) is underexplored and should be evaluated using tools like ICEfinder or PlasmidFinder. | PlasmidFinder database was used to search for plasmids (see comment 8 below).  Further exploring the role of HGT in virulence gene dissemination was out of the scope of our baseline characterization in genomic features of NOVC in Canada. However, this would be an excellent idea for exploring further genetic diversity in future studies. |
| 6. While the authors tabulate virulence genes, they fail to conduct any comparative pan-genome or synteny analyses. A deeper annotation of pathogenicity islands (e.g., VPI-1, VPI-2) using genome browsers would provide better resolution. | While these additional analyses are interesting, they would be better suited for a genome wide association study. And for the amount of isolates that we have, I do not think genome browsers would be that helpful. |
| 7. All conclusions about virulence are based on in silico predictions. There is no transcriptomic or phenotypic validation (e.g., hemolysin assays, T3SS effector secretion), which weakens claims about clinical relevance. | In silico predictions using genomics tools and pipelines still provide strong clinical relevance. Vibriowatch for example “is aimed for use by clinicians and public health workers studying cholera outbreaks, as well as for research biologists investigating V. cholerae.”  Additionally, the authors conclude that “This study has provided a snapshot of the genomic characteristics…” and that “The authors demonstrated the virulence and antimicrobial resistance potential as well as genetic diversity…” and not absolution- thus phenotypic or transcriptomic validation would not be necessary.  The authors have added a statement acknowledging deeper genomic analyses that can be done in follow-up work in the “Limitations and future directions” section. |
| 8. The AMR gene analysis is described in passing with no detailed breakdown of classes (e.g., β-lactamase variants, efflux pumps). Also, no mention is made of mobile genetic elements carrying AMR genes, which is critical in surveillance. | The presence and absence of plasmids were screened in Vibriowatch using the IncTyper tool [62]- lines 226-228 (marked up copy).  The IncTyper tool is a simple tool for identifying plasmid inc type markers in assembled genomes. The markers are sourced from the plasmidfinder database. More information can be found at <https://github.com/pathogenwatch-oss/inctyper>.  Plasmid results were added and briefly discussed lines 760-767 (marked up copy). |
| 9. The manuscript leans heavily on third-party platforms without disclosing the limitations of these tools (e.g., static databases, annotation errors). Results should be cross-validated using independent BLAST-based methods or custom HMM profiles. | Vibriowatch uses a tool called ‘VISTA’, based on BLAST, to identify virulence genes in V. cholerae genomes. More information can be found at <https://github.com/pathogenwatch-oss/vista>.  The authors have added a statement acknowledging the limitations of third party platforms in the “Limitations and future directions” section. |
| 10. The manuscript misses an opportunity to discuss how NOVC evolution compares with pandemic O1/O139 strains (e.g., clonal expansion vs. recombination-driven diversification). Phylogenetic trees should include time-calibration or molecular clock estimates. | The authors believe this would be outside the scope of this study. Our purpose was to create a baseline study of NOVC genomics in Canada that future works can be built from. “This study aims to characterize a collection of clinical and environmental NOVC collected in Canada and elsewhere to determine the virulence potential, antimicrobial resistance patterns, and population structure as well as uncover the phylogenetic relationships.”  The authors have added a statement acknowledging deeper genomic analyses that can be done in follow-up work in the “Limitations” section. |
| 11. Student’s t-test is applied to categorical variables (gene presence/absence), which violates assumptions. A more appropriate test would be Fisher’s exact or chi-square test with FDR correction. | Statistical analysis was re-done to fit categorical variables and reworded in Materials and Methods section, lines 242-245 (marked up copy). “Comparisons between two independent groups (e.g., clinical vs. environmental, extraintestinal vs. gastrointestinal NOVC infection, and serogroups) and gene presence or absence were undertaken using Pearson’s chi-squared test (Fisher’s exact test for groups of N < 5).” |
| 12. There is no accessible summary table (as a figure or supplemental file) that links isolates to year, location, lineage, serogroup, and source. This omission hinders reproducibility and reader comprehension. | Yes, supplementary table 2 includes the following headers: sample name, biosample accession, host, detailed host, isolation source, sample type, infection type, country, region, year, serogroup, biotype, serotype, fastbaps clade and lineage.  This information has also been made available within the interactive figures found here: <https://microreact.org/project/cLqNBRRphmNt1aoBeh9jmJ-fig-5> and <https://microreact.org/project/nGCx8mpZEkvX2jZW2QriJj-fig-2> . |
| 13. The classification of isolates as "clinical" or "environmental" is vague and lacks metadata evidence (e.g., case records, exposure history). Misclassification could bias virulence profile comparisons. | Clarity has been added to specify “clinical” refers to “human clinical”, and “environmental” includes “animal, food, water” sources [Lines 279-282 (marked up copy).]  Additional definitions breaking down the human clinical infections into “gastrointestinal” and “extraintestinal” were provided in lines 288-294 (marked up copy), where isolation source of bacterial samples provided reasonable inference of these infections.  Full metadata can be found in Table S2. |
| 14. Recombination filtering using PhiPack is briefly mentioned, but no recombination rate estimates or visualizations (e.g., Gubbins plots) are shown. The extent of recombination should be quantified. | PhiPack was used within the Parsnp pipeline and so while I do not know the exact number of SNPs removed, the log file suggested that all identified recombinations were successfully removed. Recombination and horizontal gene transfer is common within *V. cholerae* and these events result in many SNPs being created and thus does not reflect the true phylogenetic relationship (Liang et al., 2020. doi: 10.1128/JB.00086-20). Thus for the purpose of this study it was not necessary to quantify the extent of recombination, but rather remove these SNPs.  “Parsnp v1.2 [47] was used to reconstruct the maximum likelihood phylogenetic tree based on core genome single nucleotide polymorphisms (cgSNPs) of the 242 genomic sequences of isolates collected in Canada and using V. cholerae N16961 as the reference genome. The -x flag was used to enable filtering of SNPs in recombinogenic regions as identified by PhiPack v1.1 [48].” |
| 15. The core genome phylogenetic trees are not sufficiently annotated (e.g., bootstrap values, gene overlays). Inclusion of interactive figures (e.g., Microreact or iTOL links) is suggested but not demonstrated. | In order to maintain the clarity and resolution of these large phylogenetic trees, I’ve added links to interactive figures and fixed the annotations (e.g. bootstrap values) within Microreact, where appropriate. Links to interactive figures have been added in text under appropriate figure captions.  Phylogeny and metadata can be viewed interactively at <https://microreact.org/project/cLqNBRRphmNt1aoBeh9jmJ-fig-5> and <https://microreact.org/project/nGCx8mpZEkvX2jZW2QriJj-fig-2> |
| 16. While climate change is a plausible driver of Vibrio emergence, the manuscript repeatedly makes unquantified claims without supporting climate models or water temperature data from Canada. | While several studies support the claim that climate change is a driver in the rise of *Vibrio* bacteria (cited lines 113-115, 262-266 and 323-326, marked up copy) the authors of this manuscript do not discuss absolute certainty in relation to but rather a reasonable likelihood that climate change is a plausible driver of *Vibrio* emergence.  One global climate model published by Trinanes and Martinez-Urtaza (2021) ... “anticipated an expansion of both the temporal and spatial disease burden for Vibrio infections, in particular at high latitudes of the northern hemisphere.” … which we referenced in the introduction lines 113-115 (marked up copy). |
| 17. Genes like tdh, ctxAB, toxR are inconsistently referred to across figures and text. A consistent gene naming system should be applied (e.g., italicized for genes, capitalized for proteins). | The naming consistency of genes/proteins was reviewed and fixed throughout the manuscript text and figures. |
| 18. The manuscript misses the chance to link Canadian findings to broader One Health or global AMR surveillance initiatives (e.g., GISAID-equivalent for Vibrio, or WHO GLASS). | Global genomic surveillance initiatives for *V. cholerae* are at its infancy, especially for NOVC. PulseNet International (PNI) has a vision to “... implement globally standardized surveillance using whole genome sequencing (WGS) for real-time identification and subtyping of foodborne pathogens to strengthen preparedness and response and lower the burden of disease.”  The authors have added a statement in the “Limitations and future directions” section pointing to the vision of PNI as well as the inclusion of more environmental NOVC genomes in future works in the context of “One Health” in genomic surveillance. |
| 19. Custom pipelines like “mikrokondo” are not well-described or linked to repositories. Readers must have access to full GitHub/DOI-archived scripts and parameters for reproducibility. | Mikrokondo utilizes version control for reproducibility. The version of mikrokondo has been added to the methods and the repository can be found here: <https://github.com/phac-nml/mikrokondo/releases/tag/0.1.0> |
| 20. Although MLST types are mentioned, the manuscript doesn’t explore the evolutionary or geographic patterns of sequence types across the Canadian or global dataset using STRUCTURE or goeBURST. | The authors believe this would be outside the scope of this study. Our purpose was to create a baseline study of NOVC genomics in Canada that future works can be built from. “This study aims to characterize a collection of clinical and environmental NOVC collected in Canada and elsewhere to determine the virulence potential, antimicrobial resistance patterns, and population structure as well as uncover the phylogenetic relationships.”  The authors have added a statement acknowledging deeper genomic analyses that can be done in follow-up work in the “Limitations” section. |
| **Reviewer #2:** Greetings  Great work.  But there is one figure about the phylogenetic tree is not clear.  Kindly try to put more clear figure.  Kindly write the scientific names of bacteria by italic line style in whole manuscript.  Kind regards. | Thank you for your careful review. To improve clarity we have added interactive versions of our phylogenetic trees which can be found at <https://microreact.org/project/cLqNBRRphmNt1aoBeh9jmJ-fig-5> and  <https://microreact.org/project/nGCx8mpZEkvX2jZW2QriJj-fig-2> . Additionally, we have carefully reviewed the manuscript to ensure scientific names of bacteria are in italic line style. |
| 6. PLOS authors have the option to publish the peer review history of their article ([what does this mean?](https://track.editorialmanager.com/CL0/https:%2F%2Fjournals.plos.org%2Fplosone%2Fs%2Feditorial-and-peer-review-process%23loc-peer-review-history/1/010f0197fd0b1f8d-8a25f3cb-e1ea-4491-84dd-8d3a440a4b04-000000/Tv7Pu0RcbXiX7UImPyPKag_r0deGdD10PfsZ-sQHcg8=219)). If published, this will include your full peer review and any attached files.      If you choose “no”, your identity will remain anonymous but your review may still be made public.      **Do you want your identity to be public for this peer review?** For information about this choice, including consent withdrawal, please see our [Privacy Policy](https://track.editorialmanager.com/CL0/https:%2F%2Fwww.plos.org%2Fprivacy-policy/1/010f0197fd0b1f8d-8a25f3cb-e1ea-4491-84dd-8d3a440a4b04-000000/I896lu9PRpvU9pj4u8JPCZwO-qj2_TtohgIHzY4OlLk=219).  Reviewer #1: **Yes:** Shaban Ahmad  Reviewer #2: No |  |
| While revising your submission, please upload your figure files to the Preflight Analysis and Conversion Engine (PACE) digital diagnostic tool, [https://pacev2.apexcovantage.com/](https://track.editorialmanager.com/CL0/https:%2F%2Fpacev2.apexcovantage.com%2F/1/010f0197fd0b1f8d-8a25f3cb-e1ea-4491-84dd-8d3a440a4b04-000000/RS_PnOj8tfTr8J3jUQsyp4K3e2A3sk0wAoTNCWTUlGY=219). PACE helps ensure that figures meet PLOS requirements. To use PACE, you must first register as a user. Registration is free. Then, login and navigate to the UPLOAD tab, where you will find detailed instructions on how to use the tool. If you encounter any issues or have any questions when using PACE, please email PLOS at figures@plos.org. Please note that Supporting Information files do not need this step. | The figures uploaded were edited using PACE. |
