# Supplementary material for: Population structure and phylogenetic analysis of Vibrio cholerae non-O1/O139 by whole genome sequencing
Source: PLoS One. 2026 Mar 5;21(3):e0343306. doi: 10.1371/journal.pone.0343306 (PMC12962490; doi:10.1371/journal.pone.0343306)
Supplement: Attachment — Submitted filename: Response_to_Reviewers_auresp_2.docx [file pone.0343306.s005.docx]

**Response to Reviewers**

Dear Editor and Reviewers,

Thank you for the thorough review and constructive feedback you provided on my manuscript submitted to PLOS ONE. Your insightful comments have been invaluable in improving the quality and clarity of the manuscript. I have carefully considered each of your suggestions. Below are the responses point-by-point to journal requirements and reviewer’s feedback. We hope our revised version meets your expectations.

Thank you once again for your time, effort, and valuable contributions to the advancement of this work.

Best regards,

Taylor Wells

Corresponding Author

[taylor.m.wells@phac-aspc.gc.ca](mailto:taylor.m.wells@phac-aspc.gc.ca)

PONE-D-25-23191R1

| **Journal requirements** | **Author corrections** |
| --- | --- |
| 1. If the reviewer comments include a recommendation to cite specific previously published works, please review and evaluate these publications to determine whether they are relevant and should be cited. There is no requirement to cite these works unless the editor has indicated otherwise. | Noted. |
| 2. Please review your reference list to ensure that it is complete and correct. If you have cited papers that have been retracted, please include the rationale for doing so in the manuscript text, or remove these references and replace them with relevant current references. Any changes to the reference list should be mentioned in the rebuttal letter that accompanies your revised manuscript. If you need to cite a retracted article, indicate the article’s retracted status in the References list and also include a citation and full reference for the retraction notice. | Authors have thoroughly reviewed the reference list to ensure that it is complete and correct. |
| **Reviewer’s responses to questions** |  |
| *Comments to the author* |  |
| 1. If the authors have adequately addressed your comments raised in a previous round of review and you feel that this manuscript is now acceptable for publication, you may indicate that here to bypass the “Comments to the Author” section, enter your conflict of interest statement in the “Confidential to Editor” section, and submit your "Accept" recommendation.  Reviewer #1: All comments have been addressed  Reviewer #3: (No Response) |  |
| 1. Is the manuscript technically sound, and do the data support the conclusions?    The manuscript must describe a technically sound piece of scientific research with data that supports the conclusions. Experiments must have been conducted rigorously, with appropriate controls, replication, and sample sizes. The conclusions must be drawn appropriately based on the data presented.  Reviewer #1: Yes  Reviewer #2: Yes |  |
| 2. Has the statistical analysis been performed appropriately and rigorously?  Reviewer #1: Yes  Reviewer #2: Yes |  |
| 3. Have the authors made all data underlying the findings in their manuscript fully available?    The [PLOS Data policy](http://track.editorialmanager.com/CL0/http:%2F%2Fwww.plosone.org%2Fstatic%2Fpolicies.action%23sharing/1/010f0197fd0b1f8d-8a25f3cb-e1ea-4491-84dd-8d3a440a4b04-000000/dLg8FzZC5hK7pLh15qhg--Ye-U9X-suR2kvFIaReMaA=219) requires authors to make all data underlying the findings described in their manuscript fully available without restriction, with rare exception (please refer to the Data Availability Statement in the manuscript PDF file). The data should be provided as part of the manuscript or its supporting information, or deposited to a public repository. For example, in addition to summary statistics, the data points behind means, medians and variance measures should be available. If there are restrictions on publicly sharing data—e.g. participant privacy or use of data from a third party—those must be specified.  Reviewer #1: Yes  Reviewer #2: Yes |  |
| 4. Is the manuscript presented in an intelligible fashion and written in standard English?    PLOS ONE does not copyedit accepted manuscripts, so the language in submitted articles must be clear, correct, and unambiguous. Any typographical or grammatical errors should be corrected at revision, so please note any specific errors here.  Reviewer #1: Yes  Reviewer #2: Yes |  |
| 5. Review Comments to the Author    Please use the space provided to explain your answers to the questions above. You may also include additional comments for the author, including concerns about dual publication, research ethics, or publication ethics. (Please upload your review as an attachment if it exceeds 20,000 characters) |  |
| **Reviewer #1:** Dear authors,  I read the revised manuscript and found that your team has resolved everything and therefore I mark it for acceptance. Congratulations. | Thank you for your careful review of our revisions. We are happy to have appropriately addressed the revisions for your acceptance. |
| **Reviewer #3:** | We are grateful for your insightful comments and constructive suggestions. Our point by point responses are as follows: |
| 1. The detection of the tdh (thermostable direct hemolysin) gene in the CAD-2 lineage—which was associated with the herring egg outbreak—is a significant finding. However, the manuscript implies this gene is linked to pathogenicity similar to V. parahaemolyticus. The authors should provide a more granular analysis of this gene sequence. Specifically, what is the nucleotide or amino acid identity of this tdh allele compared to the canonical pathogenic tdh found in V. parahaemolyticus? Confirming whether this is a functional homolog or a distinct variant is crucial for substantiating the virulence potential discussion in lines 538-547. | We agree that this is a significant finding that would be worth exploring more in depth in future studies. The authors have stated that “Future studies should focus on NOVC isolates carrying the *tdh* gene to investigate the pathogenic potential of TDH in NOVC infections.”  Thank you for pointing out that the text implies that the *tdh* gene found in NOVC is linked to pathogenicity similar to *V. parahaemolyticus.* The authors have edited this section (Lines 552-556) to clarify that while *tdh* is linked to pathogenicity in *V. parahaemolyticus,* it has only been proposed to be linked to pathogenicity in non-para *Vibrio* species infections; but there is no direct evidence supporting this possibility at the time. |
| 2. In the antimicrobial resistance section (Line 725), the authors state: 'All isolates harbored at least one of the dfrA genes conferring resistance to trimethoprim.' A 100% prevalence of acquired resistance genes across diverse lineages spanning 25 years is statistically unusual for non-O1/non-O139 isolates. The authors should clarify if the specific dfrA variants detected are known intrinsic chromosomal genes in V. cholerae or legitimate acquired resistance markers. If they are intrinsic, classifying them as markers of resistance in the context of surveillance might be misleading without qualification. | Resistance to trimethoprim is acquired, not intrinsic. The authors have reworded the text to clarify this (Lines 733-738).  It is not unusual to find very high levels of antibiotic resistance in *V. cholerae* especially against drugs that have previously been used to treat cholera patients in the past. The authors have added a new statement in lines 735-738. |
| 3. The authors perform statistical comparisons between 'clinical' and 'environmental' isolates (Fig 3 and text). However, the 'environmental' category aggregates 'food' (seafood/retail), 'water', and 'animal'. As shown in Figure 3b, food isolates appear to share more virulence features with clinical isolates than water isolates do. Grouping retail seafood (which acts as a vector) with environmental water samples might dilute statistically significant differences regarding ecological adaptation vs. infectivity. I suggest a brief sub-analysis or discussion point distinguishing 'natural environment' (water/sediment) from 'food/retail' to refine the conclusion that clinical and environmental isolates differ. | Clarity has been added to the discussion (Lines 279-283) to further refine the difference between “clinical” and “environmental” isolates. |
| 4. The authors identify eight 'new' lineages (CAD1-8) and state they 'represent populations of NOVC local to Canada' (Line 519). While the phylogenetic clustering is sound based on the cited cgMLST scheme, the claim of locality should be tempered. Given the vast under-sampling of NOVC genomes globally (specifically from environmental sources in other nations), it is highly probable these lineages circulate elsewhere but simply haven't been sequenced. I recommend modifying the text to acknowledge that these are 'lineages currently unique to the Canadian dataset' rather than definitively local or endemic to Canada. | Thank you for noticing this. When the authors suggested that the eight new lineages (CAD1-8) of NOVC represented local lineages in Canada, we did not mean to suggest that these lineages only exist in Canada. You are right, it is entirely possible that these lineages are circulating elsewhere.  The authors have previously noted that the isolates used in this study does not represent the true representation of NOVC in the limitations section (Lines 830 – 835).  Additionally, we have reworded the text in the section you mentioned to clarify these lineages are “currently unique to the Canadian dataset” (Lines 521 – 525). |
| 5. While the inclusion of Microreact links is excellent for transparency, the static versions of Figure 2, Figure 4 and Figure 5 included in the manuscript are very dense and difficult to read. In Figure 2, the heatmap columns are indistinguishable without zooming in significantly, and the tip labels are illegible. I recommend providing a simplified or 'collapsed' version of the tree for the main text that highlights the CAD lineages and major clades, while moving the fully annotated, dense trees to the Supplementary Materials. | Thank you for your suggestion about providing a simplified or “collapsed” version for our tree figures. I understand it may take readers some time to navigate these figures, but the authors feel that simplifying these figures would dilute our message, and having the full (albeit dense) static versions alongside the interactive figures on microreact would be the best way to address this challenge. |
